# Proceedings of Atlanta Medical Society

**Published:** 1866-08

**Authors:** 


ARTICLE III.

*Proceedings of Atlanta Medical Society.*

ATLANTA, April 17, 1866.

Report of cases being in order—

DR. J. M. BORING reported a case of worms, succeeded by general anasarca, and finally resulting in chorea. He gave the following history of the case:

A boy 12 years old, of healthy appearance, was attacked about 12th of March, 1866, with symptoms attributed to the irritation of worms. Under the action of anthelmintic remedies, a large number of lumbricoides were discharged. In two weeks from this time, general and copious anasarcous effusion occurred, which, from the use of hydrogogue cathartics, disappeared in a few days, when symptoms of chorea made their appearance, and have, up to the present time, resisted all the means used for its relief. Blisters to the spine, quinine, iron, &c., have been used without any benefit. Seven years ago, when the boy was 5 years old, he had a similar attack, in every particular; from which he was entirely relieved in a few months, and from that time to the present attack, has enjoyed good health.

DR. J. G. WESTMORELAND thought all the symptoms detailed of the above case, resulted from the irritation of worms—that the impoverished state of the blood, consequent upon injury to the digestive functions by the intestinal irritation, led directly to the anasarcous effusion. The nervous disturbance in the form of chorea is in all probability from reflex nervous action, taking its origin in the same irritability of the bowels.

DR. O'KEEFE was of opinion that the dropsical condition was probably dependent upon irritability of the areola tissue; and while he could not satisfactorily account for this state of the tissue, or trace its connection with the intestinal irritation, to his entire satisfaction, yet he thought this a more rational explanation of the effusion, than to ascribe it to the quality of the blood itself. He considered it such a case as he preferred to call "inflammatory dropsy."

Dr. W. F. Westmoreland believed the effusion, as well as symptoms of chorea, depended on reflex nervous action—that the capillary circulation, or the areola tissue, was probably so changed by the nervous disturbance, that the escape of serum into the areola tissue was the result. He could not very well explain the particular nature of the change produced, but thought the fact not unreasonable, since it is known that the circulation and functions of other organs, are dependent upon proper nervous influence, and that the particular character of functional derangement is influenced by the particular form of nervous disturbance.

Dr. O'Keefe reported a case of delivery—a primipara—in which a considerable amount of chloroform was inhaled during the labor, with the probable effect of suspending lactation. In this case not enough milk was secreted to sustain the child, without nourishment from other sources. He mentioned this case for the purpose of aiding in the investigation instituted by Dr. Alexander some weeks since. He thought it important to settle the question, whether or not the use of chloroform in labor prevents the ordinary secretion of milk. His attention had never, till recently, been called to the subject, and the case mentioned had afforded the only opportunity of giving evidence on that point.

Dr. Boring could call to mind two cases of labor in which a mixture of chloroform and chloric ether—one part of the former to two of the latter—was given in considerable quantities without any disturbance to the process of lactation. In both instances the secretion was abundant, and in one—a premature delivery with still-born child—much trouble was caused by the amount of milk. He could remember the result, in this particular, of no case in which pure chloroform had been inhaled.

Dr. J. G. Westmoreland mentioned a case of difficult labor, in which a considerable quantity of chloroform was used, and followed by deficient lactation. So inconsiderable was the secretion, that, though the child did not survive and no other means of extracting the milk afforded, there was not enough milk secreted to give any uneasiness or trouble in any way.

Dr. Redwine had never had his attention called to this subject, but in the frequent use of chloroform in obstetrical practice, he could not remember a case from which he could give evidence in the settlement of this question.

Dr. J. G. Westmoreland reported the discovery of what he supposed to be genuine original vaccinia. He was consulted this morning, by a young man—a milk-man, for a dairy in the neighborhood of Atlanta—on account of an eruption on his hands. Upon examination, the resemblance to vaccine pustule was so striking, that inquiries were made as to whether any disease had been discovered among the cows he had been milking, and whether or not he had been previously vaccinated. The report of "little warts on the cow's tits" was made, and on examination a very imperfect cicatrix of vaccination was found upon his arm. In presence of two other medical men, the cows, from which this eruption was evidently contracted, were examined, and some six or eight, of the twenty cows examined, were evidently the subjects of what seemed to be original vaccinia*.

---

Atlanta, May 1, 1866.

Society called to order by the President, Dr. Jno. M. Johnson.

At the call for reports of cases—

Dr. J. G. Westmoreland reported a case of chronic diarrhœa—treated with creasote. A young man, aged about 22 years, had suffered for six months with chronic diarrhœa, contracted at Apalachicola, Florida. He had undergone the usual dietic management, with astringent, alterative, opiate and tonic medication, without any permanent benefit. Under the use of three or four drops of creasote suspended in half an ounce of acacia mucilage, the discharges are measurably controlled, and his strength is improving.

He mentioned this case more with the view of calling attention to the action of creasote in bowels affections, than to anything of interest connected with the disease itself. It had been the practice of some surgeons in the Confederate army, to use this remedy

* The lymph and scabs from the cows have since been introduced into the arms of non-vaccinated subjects without any effect.

in very large doses, for the cure of obstinate, acute diarrhœa and dysentery. This practice, not being general, and not having heard nor read anything of this treatment in civil practice, it is desirable that the experience of members should be given.

Dr. W. F. Westmoreland stated that at Aberdeen, Mississippi, in 1862, by the urgent request of an Assistant Surgeon, in his hospital, he consented to the administration of *heroic* doses of creasote, in a case of malignant dysentery, in which excessively painful discharges occurred at intervals of only fifteen minutes. The patient was apprised of his danger, and of the seemingly hazardous experiment that was concluded upon. His consent being obtained, a teaspoonful of creasote in a tablespoonful of water was administered. In four hours the pain was much less and the evacuations reduced to half hour intervals, when the same quantity was repeated. This portion lessened the frequency of the discharges to one every two hours, and relieved almost entirely the excessive pain accompanying them. The patient was considerably exhilarated, exhibiting symptoms of ordinary intoxication. The quantity was reduced to thirty drops, and kept up at proper intervals to keep the bowels restrained, until convalescence was perfectly established.

He had adopted the same plan with several cases in the hospital under his charge in Atlanta, with similar results in some, while in others no benefit was derived. In a few instances the symptoms were perhaps aggravated.

Dr. Connally had used, in one case, drachm doses of the creasote, with success, in obstinate diarrhœa.

Dr. Griffin gave forty to fifty drops, with very satisfactory results, in acute dysentery, and in the diarrhœa of typhoid fever.

Dr. O'Keefe mentioned a case of mania a portu, which he had treated successfully with heroic doses of tincture digitalis. He made the following report:

A patient, in hospital at Richmond, became raving with mania, from drink; so much so that the constant presence of an attendant was required to hold him in bed. Half fluid ounce of tincture digitalis was administered, and repeated in two hours, with the effect of quieting him, and in a few hours induced comfortable sleep and entire relief from the mania. The circulation was not materially affected.

Dr. Word had come to the conclusion that the profession have been greatly deceived in regard to the effects of remedies. Doses of certain agents that were once thought to be poisonous are now given with impunity. He mentioned a case in which tincture veratrum was given, by mistake, for laudanum, in the dose of twenty drops; repeated every half hour till three drachms had been taken, with no more serious effect than frequent vomiting. It was given in his absence during the night, and on making his visit the following morning, he found the patient comfortable, and very much relieved from the disease (pneumonia) under treatment.

Dr. W. F. Westmoreland had witnessed a much greater degree of tolerance in the action of veratrum. A young man of Atlanta, several years since, had taken, through mistake for paregoric, from a half to fluid ounce of tincture veratrum. Constant retching and vomiting ensued, but subsided under the action of a few ounces of brandy and half grain morphia, without any further difficulty.

The regular subject for discussion—Placenta Prævia—being announced in order—

Dr. O'Keefe moved its indefinite postponement, which was lost.

Dr. Word, from some experience in the management of obstetrical cases, with this anomalous position of the placenta, was impressed with the importance of the subject, and detailed the difficulties of delivering by the feet, through a rupture of the placenta, and of suffering the delivery to be effected by the unassisted efforts of nature. He had found a difficulty in passing the head through a rent made in the placenta, after the lower extremities and body had been forced through; and also believed that when the labor is suffered to progress naturally, there is a constant tendency for the head to be diverted, by the placenta in front of it, from the proper direction, thereby making a shoulder presentation.

Dr. J. G. Westmoreland thought, without having had much experience in the management of such cases, that the plan which would afford the best prospect for the safety of the child, without increasing the risk to the mother, should be adopted. Probably by separating a portion of the placenta, the hand may be passed up, pressing against the bleeding surface of the womb, and the delivery effected rapidly by turning, with equal safety to the mother, and a probable chance for the child.

Dr. Johnson had seen unassisted delivery effected safely to the mother; and considered that a proper understanding of the nature of the difficulty, and the theory of labor, with sound judgment, would dictate the course to be pursued in a particular case.

---

Atlanta, May 8, 1866.

Society called to order by the President, Dr. John M. Johnson.

No reports of cases made.

Discussion of the regular subject being in order, the President called on Dr. O'Keefe, one of the committee who selected it, to open the discussion on typhoid fever, the subject selected at last meeting to be discussed at this.

Dr. O'Keefe said: While it was very generally understood and acknowledged that a certain course of treatment now adopted by all, is the only safe plan of treating the disease, differences of opinion in regard to its pathology exist. The French contend that disease of the alamantry glands is essential to the existence of the fever. So certain are they of this, that when antopsy discovers no such lesion, notwithstanding the diagnosis may have been made with great confidence, it is set down as an error in diagnosis. Others, in this country, believe the primary disturbance exists in the nervous centres. He did not subscribe to either of these theories. He considered it the result of a blood poison; that the various incidental forms of disturbance—by no means uniform—are the results of this state of the blood, which, passing to all the organs, is likely to affect all.

Dr. J. G. Westmoreland was glad to find even a compromise between the views he entertained of its pathology and that of the follicular origin. And under the circumstances he was inclined to say very little upon the subject. While he could not subscribe to the doctrine just rehearsed, it was one step in the right direction, and, therefore, made no argument against it.

Dr. O'Keefe replied, asking a statement of Dr. Westmoreland's views on this question—said he had given his opinion, and if anything could be said to controvert it, he would be pleased to hear it. He thought the various forms of disturbance found in the disease most likely arises from poisoned blood, because the blood passes all parts.

Dr. J. G. Westmoreland was desirous to know what was meant by blood poison. If by it is meant a derangement of the quality of the blood, so that it does not answer the purposes of renewing the tissues and sustaining the strength and vigor of the body, and thus lead to death from this cause, he could understand that such might be the case, but that under such circumstances as surrounded typhoid fever, no such opinion could be entertained. The mere fact of the many forms of organic and functional disturbance found during the progress of the fever, does not prove that the blood must necessarily lead to them. The blood is often a vehicle by which poisons are carried to the organ of their selection, but is not necessarily changed in its properties. All poisons affect some particular organ or tissue. And while we recognize the cause of typhoid as well as remittent fever, a poison, we think that a certain part is selected by it, and is invariably poisoned or impressed by it in such manner as to derange its functions, and through it the functions of other organs dependent upon it. From the mutual dependence of their functions, it does not require a stretch of imagination to conceive that the disturbance of the functions of the nervous centres may lead to every kind of functional disturbance met with in adynamic fevers.

Dr. J. M. Johnson thought both the gentlemen preceding him were right, and both were wrong. Right in thinking that the blood and nervous centres were both the subjects of poisonous influence, and wrong in supposing (as each did for his own theory) that the seat of primary disturbance is invariably the same. He thought each of these causes, under favorable circumstances, produced the same result.

